# Toward ideal carbon dioxide functionalization

**DOI:** 10.1039/c8sc05539d

**Published:** 2019-02-20

**Authors:** Yang Yang, Ji-Woong Lee

**Affiliations:** a Department of Chemistry , University of Copenhagen , Universitetsparken 5 , Copenhagen Ø , 2100 , Denmark . Email: jiwoong.lee@chem.ku.dk

## Abstract

This *Perspective* recapitulates recent developments of carbon dioxide utilization in carbon–carbon bond formation reactions, with an intention of paving a way toward sustainable CO_2_-functionalization and its tangible applications in synthetic chemistry. CO_2_ functionalization reactions possess intrinsic drawbacks: the high kinetic inertness and thermodynamic stability of CO_2_. Numerous procedures for CO_2_ utilization depend on energy-intensive processes (*i.e.* high pressure and/or temperature), often solely relying on reactive substrates, hampering its general applications. Recent efforts thus have been dedicated to catalytic CO_2_-utilization under ambient reaction conditions, however, it is still limited to a few activation modes and the use of reactive substrates. Herein, ideal CO_2_-functionalization with particular emphasis on sustainability will be discussed based on the following sub-categories; (1) metal-catalyzed ‘reductive’ carboxylation reaction of halides, olefins and allyl alcohols, (2) photochemical CO_2_-utilization, (3) redox-neutral CO_2_-functionalization, and (4) enantioselective catalysis incorporating CO_2_ to form C–CO_2_ bonds (excluding strain mediated reactions with epoxide- and aziridine-based substrates). Recent progress in these fields will be discussed with the proposed reaction mechanisms and selected examples, highlighting redox-neutral, umpolung, and asymmetric carboxylation to postulate ideal CO_2_ functionalization reactions to be developed in the near future.

## Abstract 2

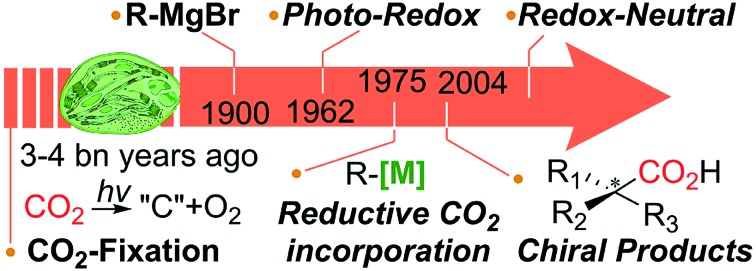
From carbon fixation, Grignard reaction, metal-catalyzed reactions and asymmetric CO_2_-incorporation, what would be the ideal CO_2_-functionalization?

## 1. Introduction

Carbon is an essential element for all living organisms, and is present in carbohydrates, amino acids, proteins, and lipids. These biomolecules are synthesized with specific selectivities controlled by the natural molecular foundry – enzymes – to sustain forms of life. The sustainability of bio- and chemical networks in living organisms is powered by the seemingly unlimited solar energy. Owing to the evolution of cyanobacteria and their photosynthesis,^1^ our planet became a unique biosphere where water was split into oxygen and hydrogen, while consuming (or fixating) CO_2_ to generate reduced organic matter.

Photosynthesis and CO_2_ fixation operate under ambient conditions; artificial photosynthesis is yet to be realized,^2^ and can ensure sustainable growth of the human civilization. The challenge lies in overcoming the thermodynamic stability and kinetic inertness of CO_2_, which possesses the highest oxidation state of carbon. Therefore, it is inevitable to employ reducing reagents (reactive metals, H_2_, electricity, and highly reducing chemicals) to overcome the intrinsic reaction barrier of CO_2_-activation, particularly to enable the reactions to be operative under mild reaction conditions.

Recently, the global society has raised concerns related to excessive energy consumption and uncontrollable anthropogenic CO_2_ emission.^3^ Although CO_2_ functionalization can provide ideal solutions, chemical reactions with CO_2_ currently suffer from low efficiency, making it impossible to mitigate the overwhelmingly large quantity of accumulated CO_2_ in the atmosphere at low concentrations.^4^ Yet, chemical recycling of carbon dioxide has been recognized as a promising supplement to the natural carbon cycle,^5^ while producing value-added fine chemicals.^6^ In this context, CO_2_ can serve as an inexpensive and non-toxic renewable C1-building block.^4^,^7^ For example, light hydrocarbons and C_1_- or C_2_-units (*i.e.* carbon monoxide, formic acid, formaldehyde, methanol, and oxalic acid) are accessible from CO_2_, mostly catalyzed by heterogeneous materials (semiconductors,^8^ zeolites,^9^ COFs,^10^ MOFs,^11^ and g-C_3_N_4_ (ref. 12)). On the other hand, homogeneous catalysis has shown remarkable potential in C–C bond formation reactions, *via* formal insertion of CO_2_ at C–H bonds. The utility of carboxylic acids and their derivatives is certainly applicable with broad interest in organic synthesis^13^ and pharmaceutical chemistry.^14^

As categorized in Table 1, catalytic CO_2_-functionalization reactions have been reviewed, particularly transition-metal catalyzed C–C bond formation reactions,^15^ carboxylation reactions catalyzed by palladium,^16^ silver,^17^ copper^18^ or copper–NHC (N-heterocyclic carbene) complexes,^19^ and nickel/iron^20^ catalysts, asymmetric CO_2_-functionalization reactions^21^ and photocatalytic CO_2_-functionalization.^22^ Other types of reactions are also tabulated to guide the readers for further reading in specific topics of interest. For example, carbonate formation reactions with epoxides and ring-strain mediated reactions,^23^ catalytic alkylation with CO_2_,^24^*etc.*, will not be discussed in this *Perspective*.

**Table 1:** A summary of recent reviews cited regarding CO_2_-utilization related subjects

Year (ref.)	Title	Keywords
2014 (ref. 3*a*)	Catalysis for the valorization of exhaust carbon: from CO_2_ to chemicals, materials, and fuels. Technological use of CO_2_	CO_2_ emission and utilization
2014 (ref. 3*b*)	Porous inorganic membranes for CO_2_ capture: present and prospects	CO_2_ capture
2001 (ref. 3*c*)	Catalysis research of relevance to carbon management: progress, challenges, and opportunities	CO_2_ emission and utilization
2007 (ref. 4)	Transformation of carbon dioxide	CO_2_ conversion
2018 (ref. 7)	Sustainable conversion of carbon dioxide: an integrated review of catalysis and life cycle assessment	Catalysis, carbon life cycle assessment
2018 (ref. 8*a*)	Cocatalysts in semiconductor-based photocatalytic CO_2_ reduction: achievements, challenges, and opportunities	Photocatalytic CO_2_ reduction
2013 (ref. 8*b*)	Photocatalytic reduction of CO_2_ on TiO_2_ and other semiconductors	
2014 (ref. 8*c*)	Photocatalytic conversion of CO_2_ into renewable hydrocarbon fuels: state-of-the-art accomplishment, challenges, and prospects	
2017 (ref. 11*a*)	The chemistry of metal–organic frameworks for CO_2_ capture, regeneration and conversion	MOFs in CO_2_ utilization
2017 (ref. 11*b*)	Metal organic framework based catalysts for CO_2_ conversion	
2015 (ref. 12*a*)	A review on g-C_3_N_4_ for photocatalytic water splitting and CO_2_ reduction	g-C_3_N_4_ in CO_2_ utilization
2018 (ref. 15*a*)	Transition metal-catalyzed carboxylation reactions with carbon dioxide	Metal-catalyzed carboxylation
2016 (ref. 15*b*)	Metal-catalyzed carboxylation of organic (pseudo)halides with CO_2_	
2018 (ref. 15*c*)	Transition metal-catalyzed carboxylation of unsaturated substrates with CO_2_	
2018 (ref. 16)	Recent advances in palladium-catalyzed carboxylation with CO_2_	
2016 (ref. 17)	Silver-catalyzed carboxylation	
2016 (ref. 18)	Copper-catalyzed carboxylation reactions using carbon dioxide	
2013 (ref. 19)	N-heterocyclic carbene (NHC)–copper-catalysed transformations of carbon dioxide	
2016 (ref. 20)	Ni- and Fe-catalyzed carboxylation of unsaturated hydrocarbons with CO_2_	
2015 (ref. 23*a*)	Recent advances in the catalytic preparation of cyclic organic carbonates	Cyclic organic carbonates
2018 (ref. 23*b*)	Catalytic strategies for the cycloaddition of pure, diluted, and waste CO_2_ to epoxides under ambient conditions	
2015 (ref. 23*c*)	Synthesis of cyclic carbonates from epoxides and carbon dioxide by using organocatalysts	
2018 (ref. 24*a*)	Catalytic reductive *N*-alkylations using CO_2_ and carboxylic acid derivatives: recent progress and developments	Catalytic alkylation
2017 (ref. 24*b*)	Utilization of CO_2_ as a C1 building block for catalytic methylation reactions	
2017 (ref. 21*a*)	Enantioselective incorporation of CO_2_: status and potential	Asymmetric functionalization
2016 (ref. 21*b*)	CO_2_-mediated formation of chiral fine chemicals	
2018 (ref. 22*a*)	Photoredox catalysis as a strategy for CO_2_ incorporation: direct access to carboxylic acids from a renewable feedstock	Photocatalytic carboxylation using CO_2_
2017 (ref. 22*b*)	Photochemical carboxylation of activated C(sp^3^)–H bonds with CO_2_	
2017 (ref. 85*a*)	Reversible hydrogenation of carbon dioxide to formic acid and methanol: Lewis acid enhancement of base metal catalysts	Formic acid and methanol derivatives
2015 (ref. 85*b*)	CO_2_ hydrogenation to formate and methanol as an alternative to photo- and electrochemical CO_2_ reduction	
2014 (ref. 85*c*)	Recycling of carbon dioxide to methanol and derived products-closing the loop	

The purpose of *this Perspective* is the following: providing a general concept of catalytic CO_2_-functionalization by exemplifying recent progress (up to 2018). Section 2 will discuss transition-metal catalysis with a hint of sustainability. Sections 3 and 4 will explore recently reported photochemical redox catalysis by utilizing synthetic dyes with the aid of pre-established transition metal catalysis, and single-electron reduction of CO_2_*via* a redox-neutral mechanism. Section 5 will focus on a handful but remarkable examples of asymmetric C–C bond formation reactions by the action of metal–chiral ligand complexes. The future perspective on ideal CO_2_-functionalization will also be discussed in the context of umpolung carboxylation, redox-neutral photochemistry and asymmetric CO_2_-activation to reduce the prevailing energy input or highly reactive species. This discussion will lead to an alternative platform for sustainable CO_2_ recycling, to mimick the natural carbon cycle by utilizing the combined knowledge in organic, inorganic, photo- and materials chemistry, and enzymatic engineering for improved carbon fixation as well.

## 2. Metal-catalyzed reductive carboxylation with halides, olefins and allyl alcohols

**Scheme 1:**
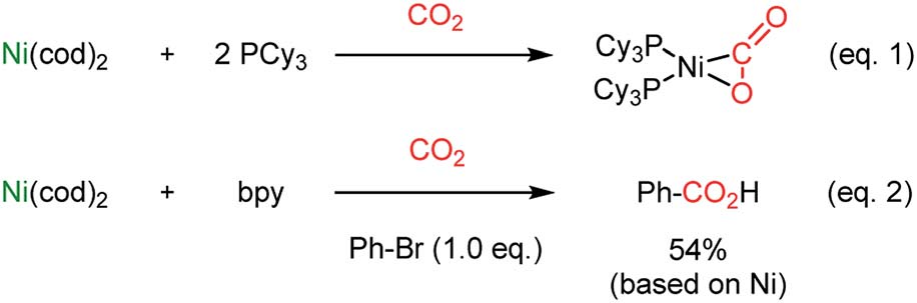
Stoichiometric CO_2_-functionalization using Ni(0).

The catalytic application of transition metals for carboxylation with CO_2_ was triggered by the seminal work by Nobile^25^ (Scheme 1, eqn (1)) and Osakada^26^ (Scheme 1, eqn (2)), where stoichiometric Ph–Ni(L)–Br (L = 2,2′-bipyridine (bpy)) participated in CO_2_ insertion at the Ph–Ni bond, affording benzoic acid as the final product.

**Scheme 2:**
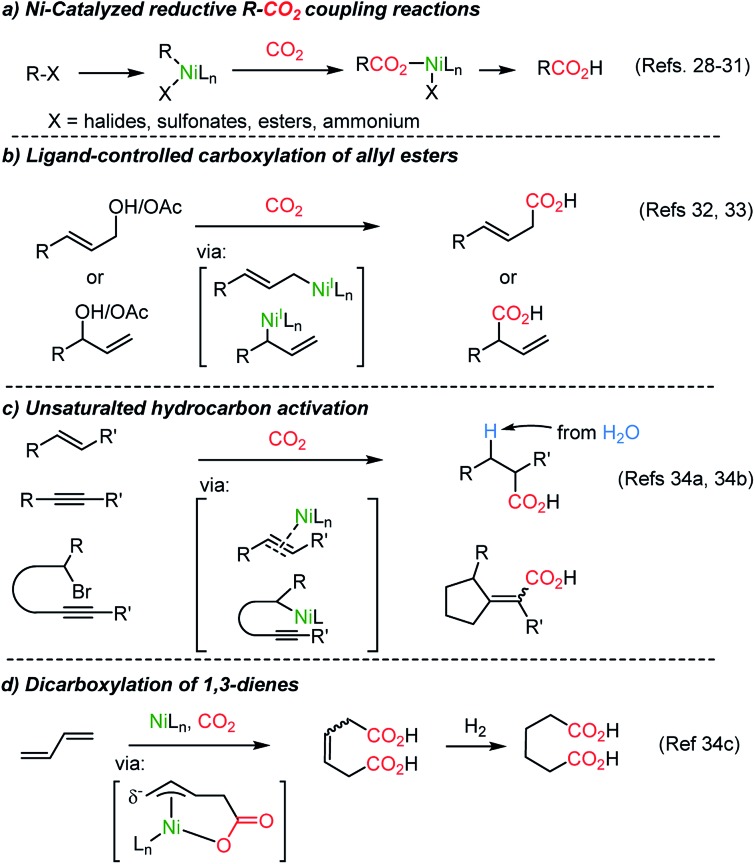
Ni-catalyzed reductive CO_2_-functionalization reactions.

The Martin group employed a Pd(ii)–Pd(0) cycle in the catalytic carboxylation reaction of aryl bromides using ZnEt_2_ as a terminal reducing reagent.^27^ This methodology was further expanded to abundant Ni(ii) catalysis by the Tsuji group,^28^ realizing carboxylation of aryl chloride with Mn powder as a reducing reagent. New reductive carboxylation reactions were developed later by the Martin group with a broad range of substrate scope, including organic halides,^29^ sulfonates,29*b* esters,^30^ benzylic ammonium salts^31^ (Scheme 2a), allyl acetates,^32^ allyl alcohols^33^ (Scheme 2b), and unsaturated hydrocarbons (Scheme 2c and d).^34^ The facile insertion of CO_2_ into R–Ni was tested with olefin substrates, enabling olefin activation without an apparent hydride donor (Scheme 2). These protocols provided a broad substrate scope and high functional group tolerance. However, it is necessary to use (over)stoichiometric amounts of reducing reagents (*i.e.* Mn, Zn, ZnR_2_, and *etc.*) to complete the catalytic cycle.

**Scheme 3:**
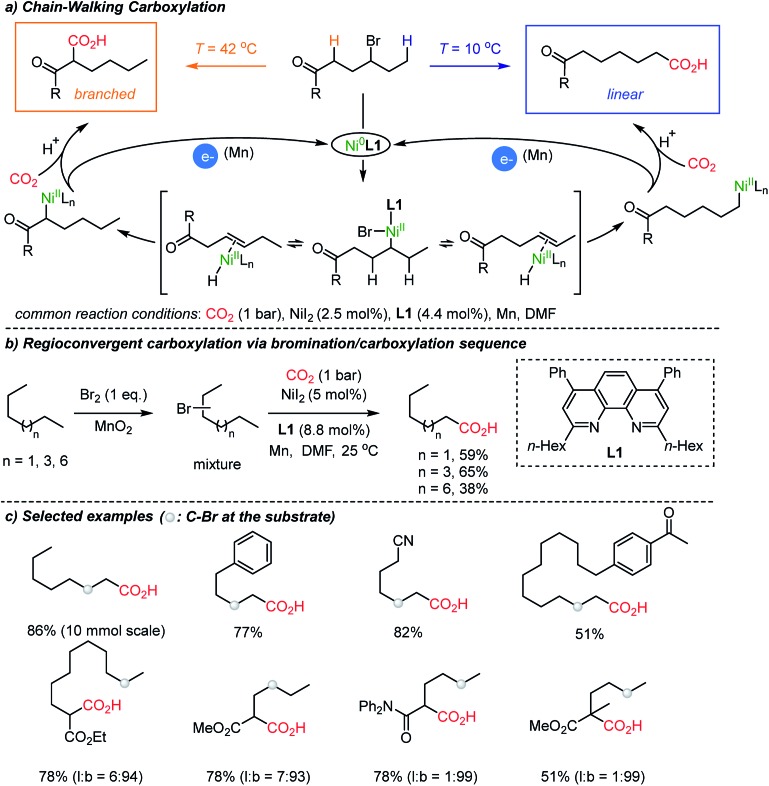
Chain-walking carboxylation of halogenated hydrocarbons.

In 2017, a breakthrough CO_2_-functionalization was reported by the Martin group proposing a ‘chain-walking’ mechanism with catalytic Ni–H species (Scheme 3).^35^ Although the β-hydride elimination is undesired in transition metal-catalyzed coupling reactions,^36^ in the proposed reaction mechanism, a chain-walking process was key to generate thermodynamically more stable species, thus contributing to the high regio- and chemoselectivity of the targeted insertion reactions.^37^ For carboxylation reactions with CO_2_, the Martin group showed temperature-controlled site-selectivity affording linear and branched carboxylated products (l : b ratios). The authors suggested a Curtin–Hammett scenario, where the reaction proceeded through common intermediates or transition states under fast equilibrium (Scheme 3a). More strikingly, the chain-walking mechanism was translated to a useful method starting from a mixture of alkyl bromides – expanding the utility of the protocol significantly. Regardless of regioisomers, linear alkanes were smoothly converted to carboxylated products under a bromination/carboxylation reaction sequence (1 atm of CO_2_). The iterative reversible β-hydride elimination/insertion reactions occurred, converging regioisomers of alkyl bromides into a single carboxylated product (Scheme 3b).

**Scheme 4:**
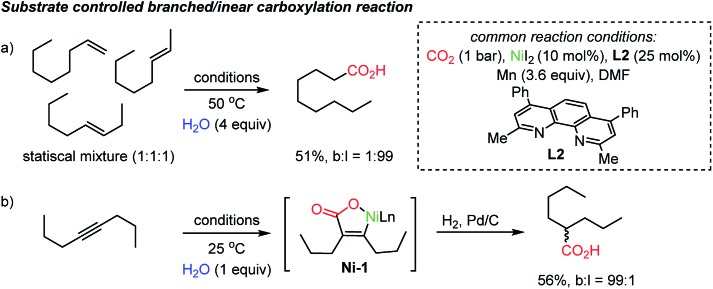
Site-selective carboxylation dictated by the degree of unsaturation.

The proposed chain-walking process with high site-selectivity represents a significant potential toward fatty-acid syntheses from bulk petroleum raw materials. In this context, the same group extended the methodology with olefin substrates, enabling carboxylation reactions in the presence of water as a proton source.34*b* In the case of alkenes, water served as a way to access metal-hydride species,^38^ namely Ni–H species, which in turn can participate in the above-mentioned chain-walking mechanism. Indeed, a linear carboxylic acid was the main product with high selectivity (b : l = 1 : 99) even from an unrefined mixture of olefin isomers (Scheme 4a). As for alkynes, however, only a branched carboxylation product was obtained (Scheme 4b). The authors proposed that the **Ni-L2** complex favored the formation of a thermodynamically more stable α,β-unsaturated nickelalactone (**Ni-1**) with internal alkynes in a CO_2_ environment. Therefore, a branched carboxylic acid was obtained with high selectivity (b : l = 99 : 1) after reduction with H_2_ and Pd/C. The ‘uni-directional’ chain-walking mechanism highlights the potential application of this process in producing added value chemicals from CO_2_ and crude industrial feedstock.

It is noteworthy that the variation of the ligand is critical in Ni-catalyzed reactions. The substituent adjacent to the nitrogen atoms in bidentate ligands (**L1** and **L2**), such as bipyridine and phenanthroline, differentiates the site-selectivity of the carboxylation reaction. High site-selectivity is a pre-requisite for many organic transformations, for example in allylic substitution reactions. Catalytic metal–ligand complexes govern chemo-, regio- and even enantioselectivity.^39^ Allyl alcohol is a substrate class with high accessibility yet low chemical utility for allylation reactions due to the apparently low leaving group ability of the hydroxide. It has been proved that *in situ* activation of allylic alcohol with ‘activating reagents’ can mediate various types of transformation,^40^ shortening the synthetic steps avoiding the preparation of activated substrates^41^ (like amines,41*a* ammonium salts,41*b* carbamates,41*c* carbonates,41*d* esters,41*e* ethers,41*f* nitro compounds,41*g* phosphates,41*h* and sulfones41*i*). For example, CO_2_ was involved in the asymmetric Pd-catalyzed direct α-allylation of ketones.40*a* The use of CO_2_ as a catalyst is noticeable although only a ‘catalytic’-amount of it would be necessary for the process.

**Scheme 5:**
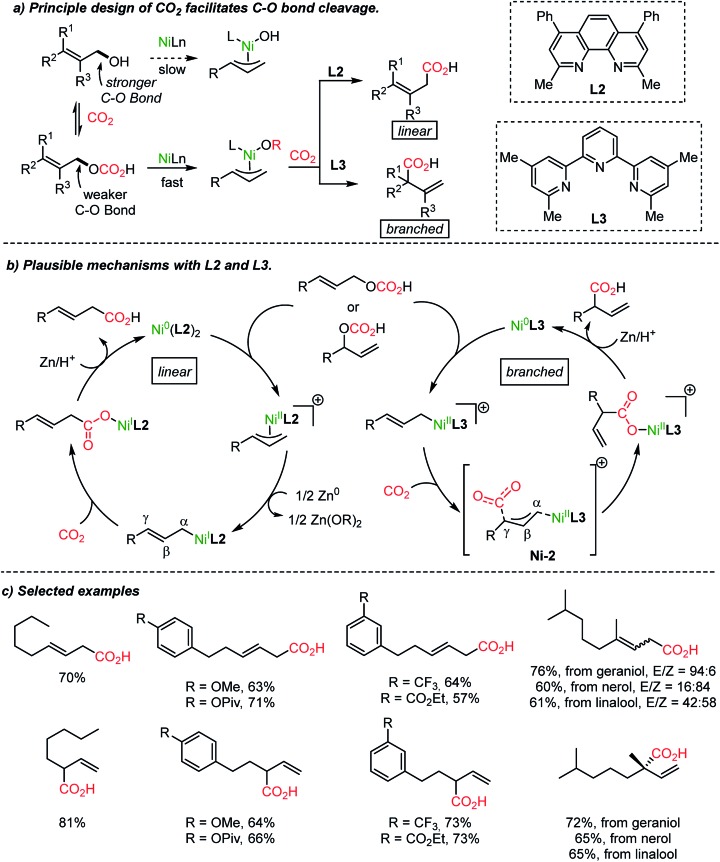
Site-selective catalytic carboxylation of allylic alcohols.

The Martin group employed CO_2_ as an activating reagent as well as a C1 source for the carboxylation of allylic alcohols to afford β,γ-unsaturated carboxylic acids (Scheme 5a).^33^ Once again, ligand-controlled selectivity was observed starting from linear or branched allylic alcohols affording high yields of linear and branched carboxylation products (Scheme 5b). The former resulted from CO_2_ insertion between the α-carbon and Ni(i) center. Alternatively, α-branched acids were obtained when the tridentate ligand **L3** was employed. The critical role of the ligands was rationalized by stoichiometric studies of active Ni**L2** or Ni**L3** species in the absence of Zn metal (yields: linear, 0%; branched 73%). The transition-state, **Ni-2**, was proposed for the nucleophilic attack from the γ-carbon of an η^1^-allyl Ni(ii) intermediate to CO_2_. Also, a six-membered cyclic conformation can be suggested, similar to the reported nucleophilic addition of Pd-(π)allyl intermediates to CO_2_ or carbonyl substrates.^42^,^43^ The utility of the reaction was further verified by producing useful intermediates for the synthesis of γ-lactone-based bioactive compounds.^44^

**Scheme 6:**
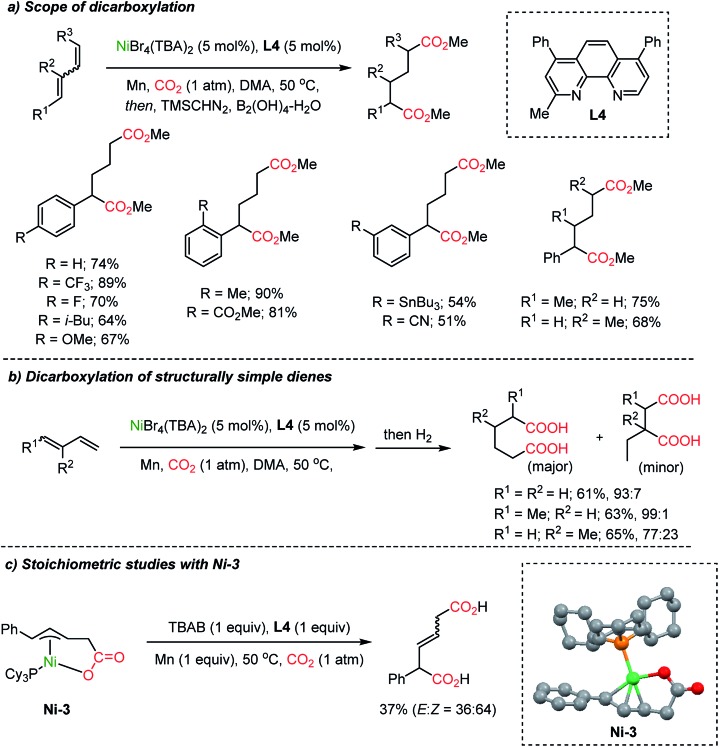
Ni-catalyzed dicarboxylation of 1,3-dienes and a mechanistic study.

Dienes, abundant and accessible chemical feedstocks, have the same oxidation states as allylic alcohols. However, activation of dienes and conjugated olefins poses a great challenge. Recently, Ni-based catalysts were evaluated for a catalytic carboxylation reaction of dienes toward carboxylated or dicarboxylated products in stoichiometric amounts of a Ni(0) complex.^45^ Although limited only to activated substrates, alkynes^46^ and silylallenes^47^ were transformed to the desired dicarboxylated products. The Martin group successfully implemented a catalytic dicarboxylation reaction for 1,3-dienes with high site-selectivity (up to 90%), to furnish diesters (Scheme 6a).34*c* Various functional groups were tolerated including heterocycles, organotin, nitrile, and esters. Structurally simple dienes such as butadiene, isoprene, and piperylene – major byproducts of steam cracking in ethylene production plants – were converted to the corresponding terminal diacids with excellent site-selectivity in moderate yields (up to 65% yield, 99 : 1 selectivity, Scheme 6b). Single crystal structure analysis determined the formation of monocarboxylated η^3^-Ni nickelalactone (**Ni-3**). The corresponding dicarboxylation product could be obtained when **Ni-3** was treated with CO_2_ under optimized reaction conditions (also see Mori group's work45*c*), shedding some light on the reaction mechanism (Scheme 6c).

The transition metal-catalyzed carboxylation reactions of the above-mentioned recent examples showed unprecedented catalytic performances with a variety of substrates, yet they require stoichiometric reducing reagents to sustain the catalytic cycle. Certain improvements have been attempted by utilizing insoluble reducing reagents (Mn, and Zn powder) replacing highly reactive RMgX, Et_2_Zn, or AlMe_3_. In an ideal CO_2_ functionalization process, a redox-neutral mechanism would be more desirable,^48^ where no additional oxidants or reductants are required. In this context, the next two section will describe reactions utilizing photocatalysts, demonstrating sustainable light-induced chemical reduction reactions, mimicking photosynthesis.

## 3. Photocatalytic carboxylation with CO_2_

**Fig. 1:**
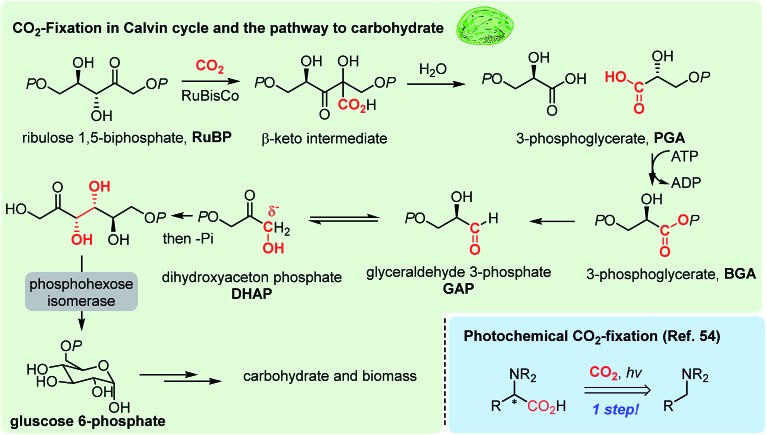
Natural photosynthesis and an example of artificial photosynthesis.

Photosynthesis is the master process in the realm of CO_2_-functionalization, as it is called CO_2_-fixation. This ideal process operates *via* multi-step electron transfer and chemical transformation reactions,^49^ resulting in somewhat limited CO_2_-fixation efficiency, constraining the capacity of nature's carbon cycle (Fig. 1 highlighted in green).^50^ Recent efforts in enzymatic engineering in chemical biology for *in vitro* CO_2_ fixation^51^ would potentially lead to enhanced photosynthesis.^52^

Solar energy obviously represents one of the most promising and limitless energy sources, which can be harnessed using a photosensitizer. In the late 1970s, seminal studies were reported regarding photocatalytic CO_2_ reduction by the Tazuke, Fujishima, Honda, and Lehn groups,^53^ which formed the basis of the modern photoredox activation of CO_2_. Further developments in photocatalysts played a significant role in CO_2_ reduction reactions mainly targeting industrial feedstock molecules, such as carbon monoxide, methanol, methane and formic acid.^8^ In this regard, artificial CO_2_ functionalization reactions have shown elegant modes of action in C–C bond formation reactions.^22^ For example, a photochemical CO_2_-fixation provided α-amino acid derivatives in a one-step reaction (Fig. 1 bottom right^54^). The key to the success of this field will be to maintain mild reaction conditions to conserve complex molecular structures of products, while providing appropriate reduction potential for the reductive CO_2_-functionalization.

**Scheme 7:**
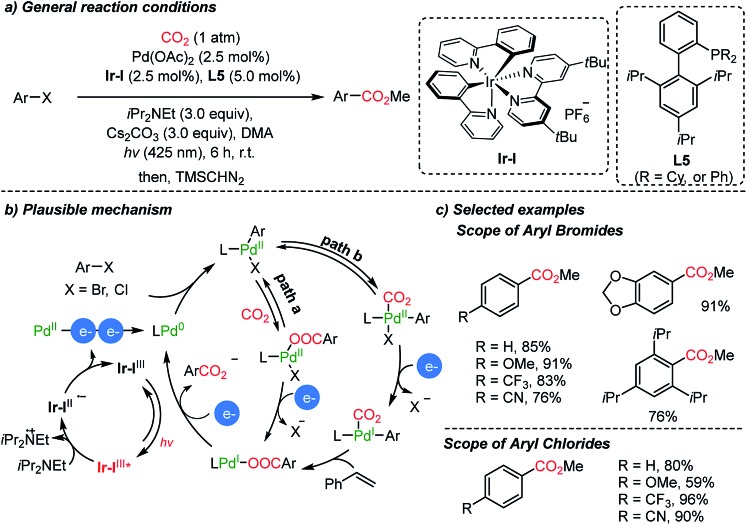
Carboxylation of aryl halides by Pd/Ir dual catalysis.

The Iwasawa group demonstrated a dual catalytic system with a Pd/Ir-photocouple for carboxylation reactions of aryl halides in the absence of metallic reducing reagents (Scheme 7).^55^ Hünig's base (3 eq.) served as a sacrificial electron donor in photoredox cycles, generating Pd(0)-complexes in the proposed catalytic carboxylation cycles (Scheme 10b). Although Ar–Pd(ii)–Br(XPhos) possesses a high reduction potential (–2.28 V, *vs.* Fc/Fc^+^), a new peak at –1.4 V was observed from cyclic voltammetry (CV) measurements. The coordination of CO_2_ on Pd might influence the redox chemistry of the metal complex, therefore reducing the required reduction potential. In addition to the common insertion of CO_2_ into the active Pd(ii)–C bond, the authors suggested the formation of two intermediates, a Pd(i)- or Pd(ii)–CO_2_ complex (Scheme 7b, path b). After methylation with TMSCHN_2_, various carboxylic acid esters were obtained including a sterically hindered acid (*i.e.* 2,4,5-triisopropyl carboxylic acid methyl ester).

**Scheme 8:**
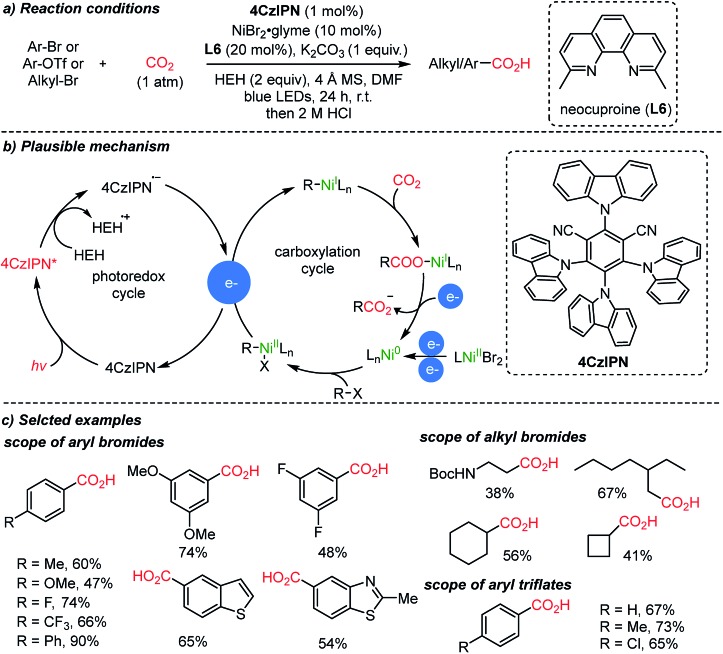
Photoredox cycles and carboxylation cycles in a co-catalysis system.

Starting from simple feedstocks, Ar–Br and alkyl–Br, the König group reported visible light-induced carboxylation mediated by nickel catalysts (Scheme 8).^56^ The plausible reaction mechanism could be divided into two distinct catalytic cycles. The first one involved a one electron delivery to a Ni(ii) or Ni(i) complex from the anion radical (**4CzIPN**˙^–^). Hantzsch ester (**HEH**, 2 equiv. required) was used as a terminal reducing agent in the presence of a reducing excited sensitizer (4Czlpn*) and light (left circle, Scheme 8b). Second, the oxidative addition to a Ni(0) complex was suggested, which undergoes reduction and then an insertion reaction with CO_2_ (right circle, Scheme 8b). The catalytically active Ni(0) species can be regenerated from Ni(i) with electron sources produced from the left circle.

**Scheme 9:**
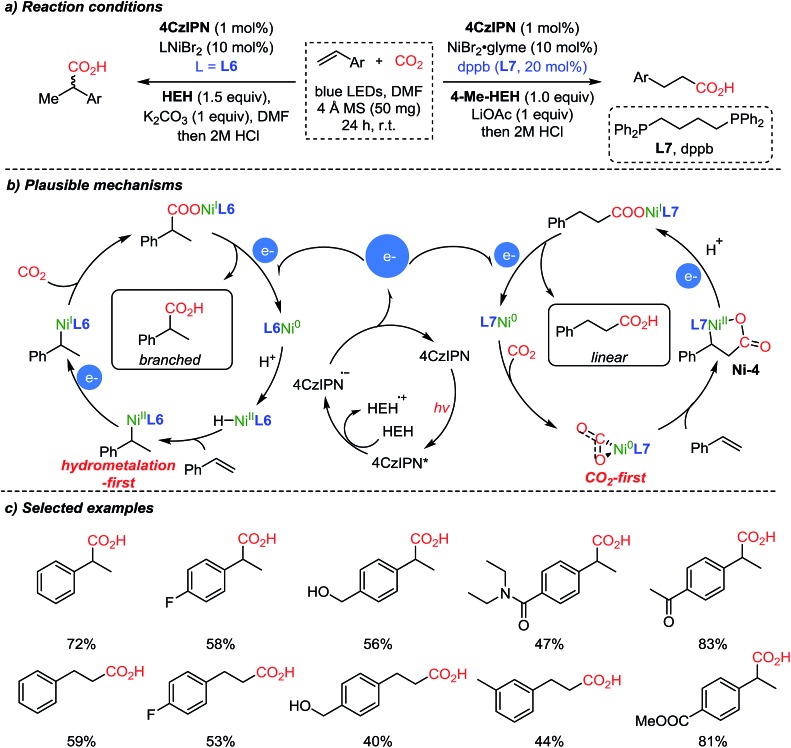
Site-selective photocatalytic carboxylation controlled by ligands.

The same group expanded the dual catalysis strategy to the carboxylation of styrenes,^57^ affording Markovnikov (branched) or anti-Markovnikov (linear) products selectively controlled by the choice of the ligand (Scheme 9). The suggested reaction mechanism explained that the observed chemoselectivity (branched/linear) was controlled by the different ligands (**L6**, neocuproine and **L7**, dppb). According to DFT calculations, Ni(0) species with the more sterically demanding ligand dppb (**L7**) tend to coordinate with CO_2_, forming a 5-membered nickelalactone (**Ni-4**) with styrene. With the less hindered neocuproine ligand (**L6**), the reaction proceeds *via* hydrometalation of styrene to afford Ni(ii), which is subsequently reduced by the catalytic action of the photosensitizer (**4CzIPN**, Scheme 9b). The electrons generated from the photocatalytic cycle are used to reduce Ni(ii) or Ni(i) to Ni(0), which can diverge to the hydrometalation step (left) and CO_2_ activation step (right) to generate branched and linear products respectively while completing the catalytic cycles.

**Scheme 10:**
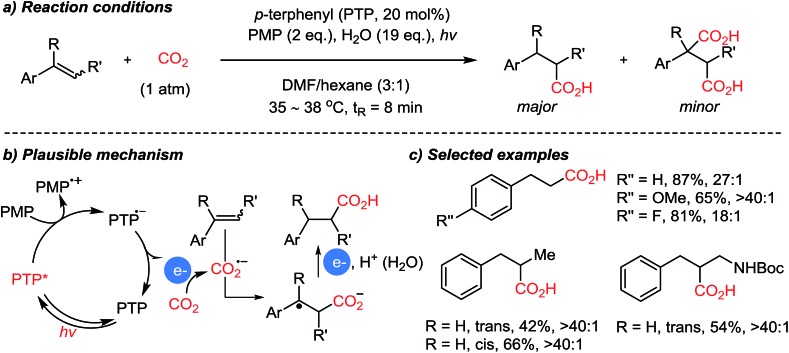
Photocatalytic direct β-selective hydrocarboxylation of styrenes.

The Jamison group reported styrene functionalization reactions in a CO_2_ atmosphere to generate β-aryl carboxylic acids (Scheme 10).^58^ In this case PMP (1,2,2,6,6-pentamethylpiperidine) was employed as a sacrificial organic electron donor, while utilizing water as an additive under modified reaction conditions. Although it is unclear, the addition of water induced high selectivity toward the mono-carboxylated product compared to other tested hydride or proton donors. The suggested reaction mechanism shows that the carboxylation with CO_2_˙^–^ results in the formation of a stabilized benzylic radical (*E*^0^ = from –1.82 to –0.71 V *vs.* SCE). Therefore, further reduction is feasible leading to the generation of carboxylated benzylic anion species, which could be protonated upon addition of water.

Photoactivation of organic substrates has been a successful transformation with high chemoselectivity to produce Markovnikov (branched) or anti-Markovnikov (linear) carboxylic acids. Also, the Murakami group reported a carboxylation reaction with α-alkyl ketones and CO_2_*via* a Norrish type II activation mechanism.^59^ The carboxylation reactions at toluenyl carbon were also conducted in natural sunlight at ambient temperature with good isolated yields of the desired products. The authors suggested an energetically feasible [4 + 2]cycloaddition reaction by DFT calculations, which was determined by the thermal reaction of benzocyclobutenol to generate an *o*-quinodimethane intermediate.

**Scheme 11:**
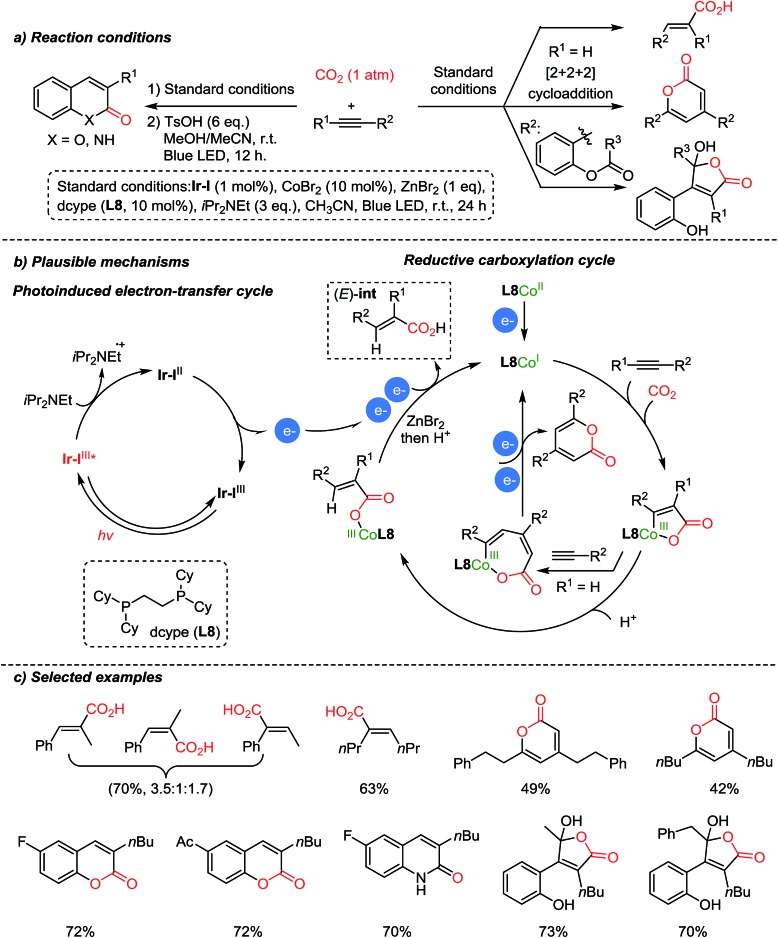
Carboxylation of alkynes by Co/Ir dual catalysis.

Recently, Hou *et al.* reported carboxylation reactions of internal and terminal alkynes promoted by Co/Ir dual catalysis (Scheme 11).^60^ The authors proposed that the reaction proceeded *via* functionalization of alkynes to generate an (*E*)-Co–CO_2_ complex which is an intermediate for various products – carboxylic acids, pyrones, α,β-unsaturated γ-lactones, coumarins, and 2-quinolones, by sharing a common intermediate, (*E*)-**int** (Scheme 11a). Pyrones were formed through a formal [2 + 2+ 2] cycloaddition with terminal alkynes (R^1^=H). In the case of internal alkynes, pharmaceutically vital heterocycles such as coumarins and 2-quinolones were obtained with high selectivity. The suggested mechanism proceeded *via* intramolecular cyclization of acrylic acid intermediates. The *E*/*Z* isomerization of acrylic acid was confirmed by control experiments with (or without) the Ir-photoredox catalyst under irradiation (or in the dark). Also, this newly developed carboxylation/acyl-migration cascade reaction is feasible for alkyne difunctionalization, highlighting its utility in the field of light-driven CO_2_-fixation.

**Scheme 12:**
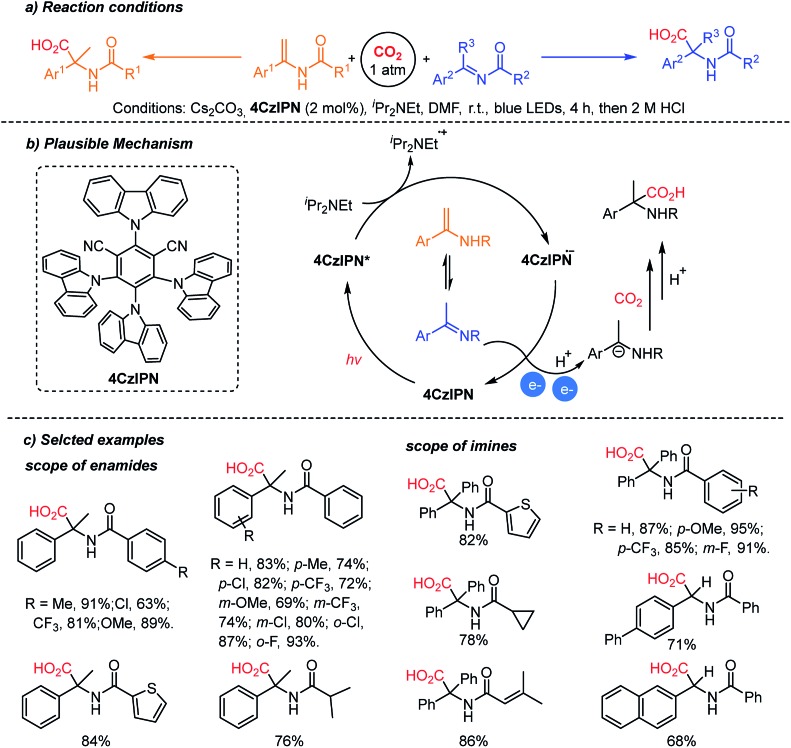
Photocatalytic hydrocarboxylation of enamides and imines.

The Yu group reported the photocatalytic hydrocarboxylation of enamides and imines to afford α-amino acids with excellent chemo- and regio-selectivity (Scheme 12).^61^ The pre-equilibrium of enamides and imines was combined with photocatalytic reduction. Despite the inherent nucleophilicity of enamides, kinetic studies indicated that the imines underwent the desired hydrocarboxylation faster than the competitive β-carboxylation reaction. The authors proposed an umpolung reaction of the α-amino carbanion under metal-free conditions. The carboxylated products were obtained with a broad substrate scope regardless of the electronic and steric properties of substituents. In addition, the enamide and imine starting materials were equally effective, confirming the fast pre-equilibrium before the reduction/carboxylation steps.

**Scheme 13:**
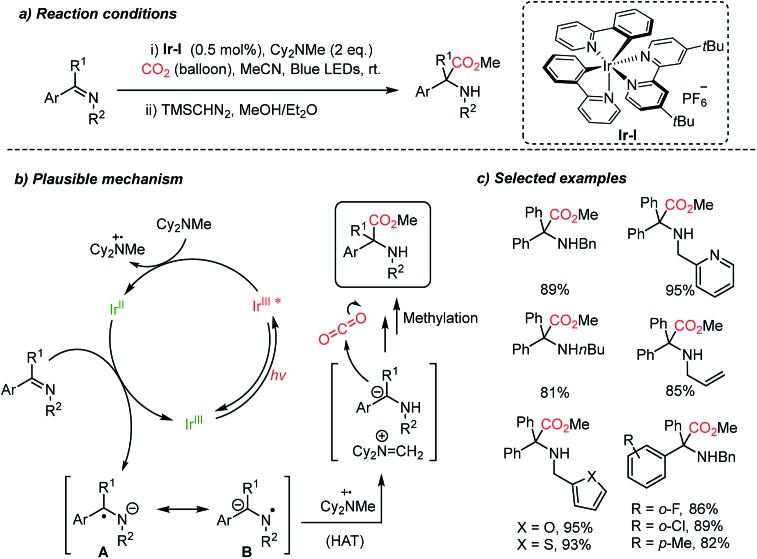
Photocatalytic carboxylation of ketimines.

Very recently, the Walsh group presented photocatalytic carboxylation of benzophenone-derived ketimines by employing an Ir-complex (**Ir-I**) under mild conditions (Scheme 13).^62^ The radical anion was generated by single electron transfer (SET) from [**Ir-I**]* to ketimines, which was facilitated by the coordination between the imine and Cy_2_MeN˙^+^.^63^ Spin density calculation was carried out to evaluate the radical anions (**A**, **B**) suggesting that the carbon atom was more negatively charged than the nitrogen atom (spin density, radical probability on C: 0.05–0.18 and N: 0.37).^64^ Subsequently, the more reactive *N*-centered radical species abstracts a hydrogen atom from Cy_2_MeN˙^e^ to form an α-amino carbanion and an iminium cation [Cy_2_N=CH_2_]^+^*via* an umpolung reactivity (Scheme 13b). The carbanion then undergoes nucleophilic addition to CO_2_ affording the desired carboxylation product. The obtained α,α-disubstituted α-amino acid shows potential application of the protocol in asymmetric synthesis to generate quaternary stereogenic centers, which are often difficult to control.^65^

**Scheme 14:**
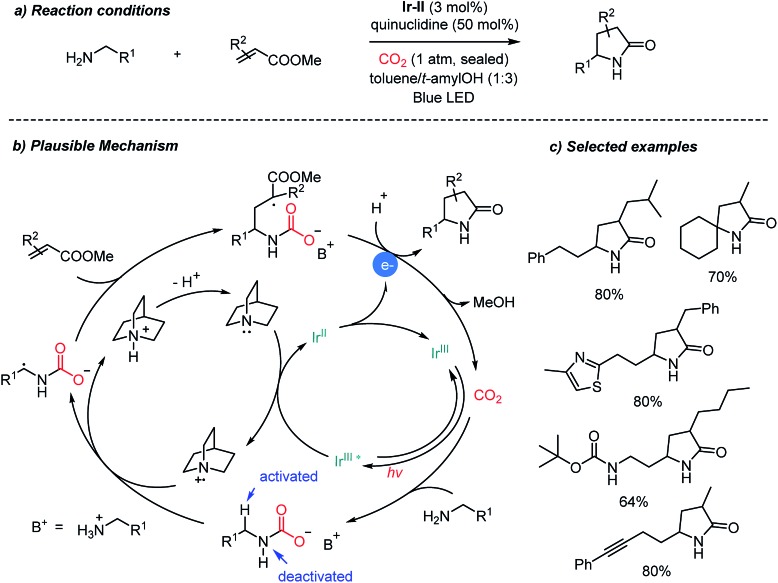
Catalytic application of CO_2_ for photocatalytic α-alkylation of primary amines.

Direct carboxylation of imines and amines with CO_2_ represents a very promising pathway to afford α-amino acids, especially those promoted by photoredox catalysts as shown above (Schemes 12, 13 and 17). Compared to tertiary amines, however, α-functionalization (*i.e.* α-carboxylation) of primary amines still remains a great challenge due to the lower reactivity of the α-C–H bond. Besides carboxylation reactions, CO_2_ has been used as an activating group,^33^,^40^ a directing group^66^ and a protecting group^67^ in organic synthesis. Ye *et al.* recently reported the photocatalytic α-alkylation of primary amines to yield γ-lactams with CO_2_ as a temporary activator and as a protecting group (Scheme 14).^68^ Various α,β-unsaturated esters were tolerated in the presence of an **Ir-II** photosensitizer. Quinuclidine was employed as a sacrificial electron donor. According to the suggested reaction mechanism, CO_2_ was regenerated after releasing lactam products *via* an intramolecular cyclization reaction. The *in situ* carbamate formation reaction suppressed the reactivity of primary amines while increasing the reactivity of α-C–H bonds according to the computational studies. The generation of the α-radical of the substrate is highly intriguing due to the potential applications toward various electrophiles and radical–radical coupling reactions. Furthermore, the use of tertiary amines as a base will enable a potential asymmetric catalysis to afford enantioenriched products.

This section summarizes recent progress in photo-CO_2_-functionalization without strong metallic reducing agents. Instead, an organic sacrificial electron source or a reducing reagent was employed (*i.e.* triethylamine, piperidine, Hünig's base and Hantzsch esters) in the presence of photocatalysts with an appropriate reduction potential to complete the catalytic cycles. Various types of substrates underwent C–CO_2_ bond formation reactions to provide unique molecular structures under ambient photosynthetic conditions (low CO_2_ pressure, and accessible light sources). However, there is still plenty of room to develop more elegant methodologies in terms of sustainability. The next section will discuss redox-neutral carboxylation without external reductants.

## 4. Recent developments in redox-neutral CO_2_-functionalization

**Fig. 2:**
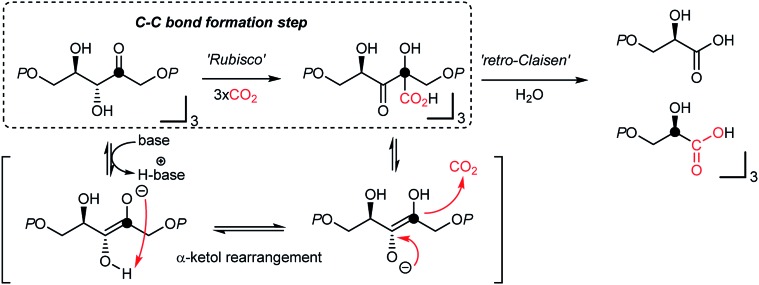
Calvin–Benson–Bassham (CBB) cycle at the RubisCo enzyme reaction center. Note that the C–CO_2_ H bond formation reaction occurs at the ketone functional group, and the two glyceric acid products are enantiomerically enriched.

It is thought that catalytic carboxylation of non-activated organic substrates would be an ideal approach to CO_2_-utilization, avoiding reactive organometallic reagents (RMgX, RLi, R_2_Zn, R_4_Sn, *etc.*). For example, solar energy provides chemical reduction potential to enable CO_2_ conversion in the Calvin cycle, where actual CO_2_-fixation and C–CO_2_ bond formation reaction occur under mild conditions *via* an α-ketol rearrangement (Fig. 2).^50^,^69^ This “enantioselective” CO_2_-fixation process generates a new C–C bond while creating additional stereogenic center(s) *via* a redox-neutral pathway. Accordingly, recent progress in photo-redox catalysis offers a promising platform to develop sustainable CO_2_ utilization reactions under mild conditions in the absence of additional reducing reagents.^54^,^70^ When combined with practicability and scalability, redox-neutral CO_2_-functionalization strategies will provide a tangible scenario of sustainable artificial carbon fixation.

The following examples in this section represent their redox-neutral reaction profile in terms of the proposed reaction mechanisms – no terminal reducing or oxidizing reagents. Despite the fact that these reactions require activated substrates or radical initiators or a strong base, the generation of C–CO_2_ bonds with CO_2_ is a remarkable step toward truly ideal CO_2_-functionalization. Keeping in mind that solar energy might be the only and truly sustainable energy source, a few examples of redox-neutral photocatalytic CO_2_-functionalization reactions are also highlighted in this section.

**Scheme 15:**
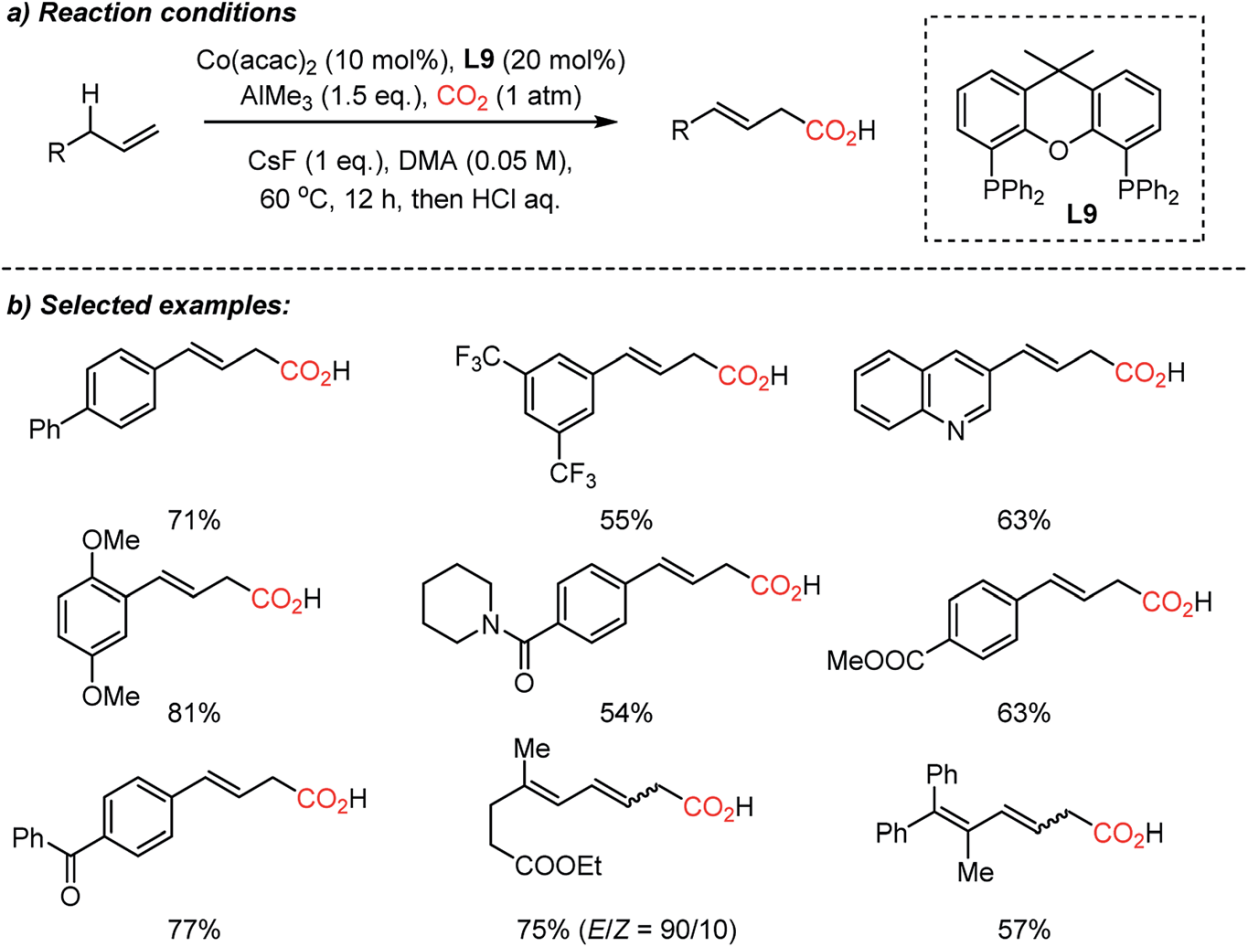
Cobalt-catalyzed direct carboxylation of allylic C(sp^3^)–H bonds.

The Sato group recently reported a direct carboxylation reaction at the allylic C(sp^3^)–H bond (Scheme 15).^71^ The use of the AlMe_3_ – non-nucleophilic base – was ascribed to the initial generation of catalytically active Co(i) species, therefore the catalytic cycle is free from an external reducing reagent. The carboxylation reaction of allylarenes and 1,4-dienes was proven to be effective with a nucleophilic η^1^-allyl-Co(i) catalyst after intensive screening of transition metal catalysts such as Cr(ii), Mn(i), Fe(iii), Rh(i), Ir(i), Ni(ii) and Cu(i). The role of the ligand was critical; Xantphos (**L9**) showed high selectivity without the formation of isomerization or methylation byproducts by the use of AlMe_3_. Various terminal alkenes were smoothly converted to β,γ-unsaturated acids with excellent functional group tolerance, including amides, esters, and ketones. The authors suggested that the presence of the low-valent Co(i)-complex was the key to the successful carboxylation reaction with high selectivity. This protocol expands upon the scope of carboxylation to C(sp^3^)–H bonds, which represents atom- and step-economic approaches to construct molecular complexity by incorporating CO_2_.

**Scheme 16:**
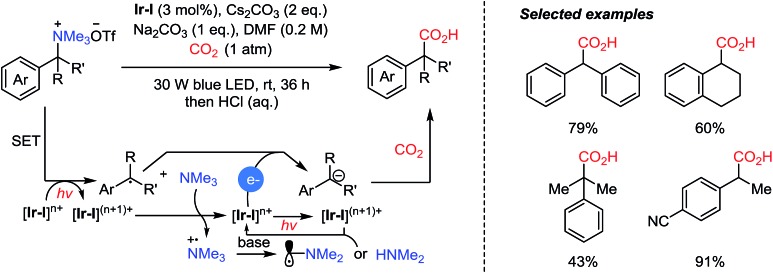
Photocatalytic carboxylation with a built-in reductant as the electron donor.

Very recently, the Yu group reported photocatalytic carboxylation of tetraalkyl ammonium salts *via* C–N bond cleavage (Scheme 16).^72^ Trimethylamine was generated *in situ* by single-electron transfer (SET) from the excited **Ir-I** to the substrates. In turn, the resulting active **Ir-I** species could be reduced by the tertiary amine. Afterwards, carbanions undergo a carboxylation reaction after another SET step between the excited photoredox catalyst and the alkyl radical. The authors suggested that the oxidized trimethylamine was transformed to amine species, like α-radical [Me_2_NCH_2_˙], or dimethylamine after hydrolysis. As electron donors, trimethylamine and dimethylamine accounted for 2 equivalents of reducing reagents required to complete the catalytic cycle. This built-in reductant was generated and demonstrated carboxylation reactions without additional reducing reagents, compared to Ni-catalyzed reductive carboxylation of benzylic C–N bonds.^31^

**Scheme 17:**
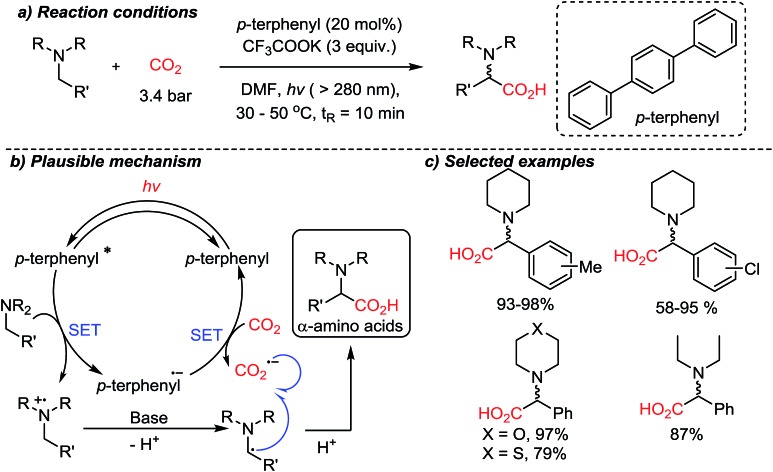
Photoredox CO_2_-activation to access α-amino acids using a *p*-terphenyl photosensitizer.

In the above-mentioned cases, organic amines act as sacrificial electron donors, where the resulting radical cation trialkyl amines have dramatically reduced p*K*_a_ at the α-protons.^73^ In the presence of a base, a deprotonation reaction would generate an amine with an α-radical, which can couple with other reactive species. The single-electron reduction of CO_2_ to CO_2_˙^–^ is in general a rate-determining step due to the high reduction potential (–2.21 V *vs.* SCE (saturated calomel electrode) in DMF (*N*,*N*-dimethylformamide)).^74^ A viable C–C bond formation reaction with CO_2_˙^–^ and amine based α-radicals would afford α-amino acids as the product. This was realized by the Jamison group demonstrating a metal-free photoredox conversion of CO_2_ (Scheme 17).^54^ An organic sensitizer, *p*-terphenyl, mediated single electron transfer reactions (reduction potential: –2.63 V *vs.* SCE in DMF) to perform the suggested one-electron reduction of CO_2_, providing α-amino acids in the absence of additional reducing reagents. Various aryl-substituted α-amino acids were prepared in good to excellent yields. The convenience of continuous flow chemistry^75^ was an added benefit of the photocatalysis to provide essential synthetic building blocks from carbon dioxide. The generation of CO_2_-radical anion is highly attractive, considering its vast application potential in organic synthesis for carboxylation reactions. This photocatalysis mediated by *p*-terphenyl showed promise toward metal-free CO_2_-functionalization *via* a single-electron reduction mechanism in terms of atom-economy (redox-neutral), feasibility (continuous flow setups), and utility of the final products (α-amino acids) containing stereogenic centers.

**Scheme 18:**
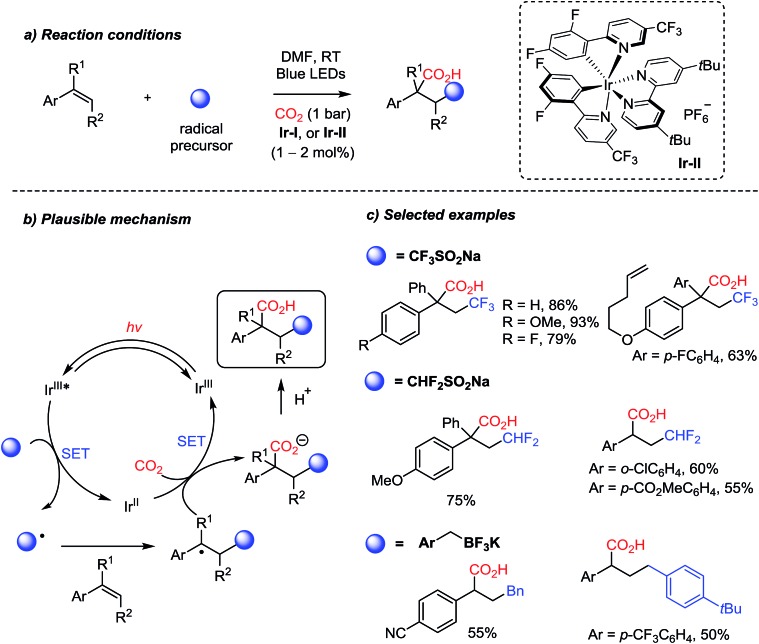
Structural diversity of carboxylation with radical initiators.

Owing to the recent developments in organic photosynthesis and photosensitizers,^22^,^76^ unprecedented reactivity patterns were achieved with CO_2_ as a C1 source. For example, the Martin group showed photocatalytic dicarbofunctionalization of styrene derivatives initiated by radicals under mild reaction conditions, where stabilized benzyl carbanions react with CO_2_ (Scheme 18).70*a* Various radical initiators, such as trifluoro- and difluorosulfonates, and trifluoroborate salts, were proven to be effective under photochemical reaction conditions. The photocatalytic redox cycle was mediated by an Ir-complex (**Ir-II**). This protocol provides two new C–C bonds with a stereogenic center in the absence of additional stoichiometric reducing reagents. Trisubstituted alkenes were also employed to afford carboxylic acids with a quaternary stereogenic center. The convenient introduction of the (di)trifluoromethyl group highlights potential applications of radical carboxylation reactions in drug discovery and pharmaceutical industry.^77^

**Scheme 19:**
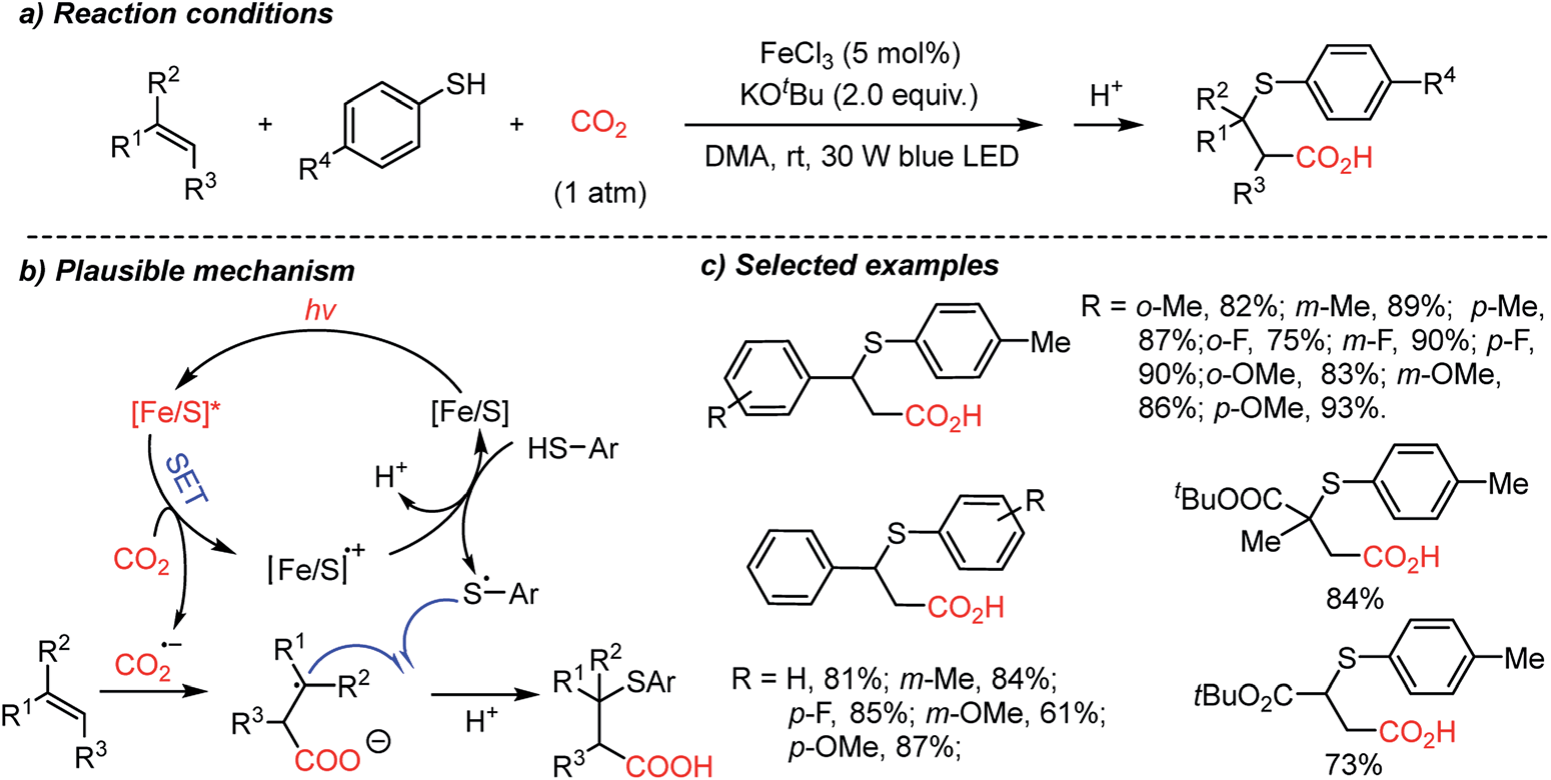
Fe–S catalyzed thiocarboxylation induced by visible light.

The Yu group developed the first thiocarboxylation of styrenes by using an Fe/S complex as the photosensitizer (Scheme 19).70*b* Various β-thioacids were synthesized selectively with different regioselectivities from the previous protocol (Scheme 18). Mechanistic studies revealed that single-electron reduction of CO_2_ can be initiated by the excited Fe/S complex, yielding the CO_2_ radical anion (CO_2_˙^–^). This radical intermediate was trapped subsequently by an alkene substrate to generate a stabilized alkyl radical, which led to anti-Markovnikov regioselectivity. Thiolation of alkyl radicals was mediated by the [Fe/S] radical cation, highlighting the application potential of the methodology in the synthesis of β-thioacids – an intermediate for the antidepressant drug thiazensim.^78^ Also, considering the Fe- and S-rich environment in the prebiotic era, the presented reaction could help us to rethink the CO_2_ chemistry in the primordial soup, potentially affording complicated photoredox reactions with CO_2_ to furnish chiral molecules.

The progress in redox-neutral CO_2_ functionalization showed elegant reaction mechanisms operating under mild conditions, for example, *via* CO_2_ insertion into metal–carbon bonds or CO_2_˙^–^ captured by activated substrates. This represents a promising and ideal mode of action, whereby no additional sacrificial redox agents were applied to construct multiple C–C, and C–X bonds. Thus, high atom economy and step-efficiency are expected in constructing molecules with CO_2_ as a non-toxic C1 source, boosting research in CO_2_-utilization from recently developed dicarbofunctionalization.^79^ Meanwhile, the structural diversity of recent CO_2_-functionalization reactions shows the significant potential of CO_2_ in asymmetric synthesis and catalysis. Further investigations on the asymmetric activation of CO_2_ and its utilization in CO_2_-functionalization will allow us to achieve higher values of products while recycling CO_2_.

## 5. Asymmetric catalytic carboxylation with CO_2_

**Scheme 20:**
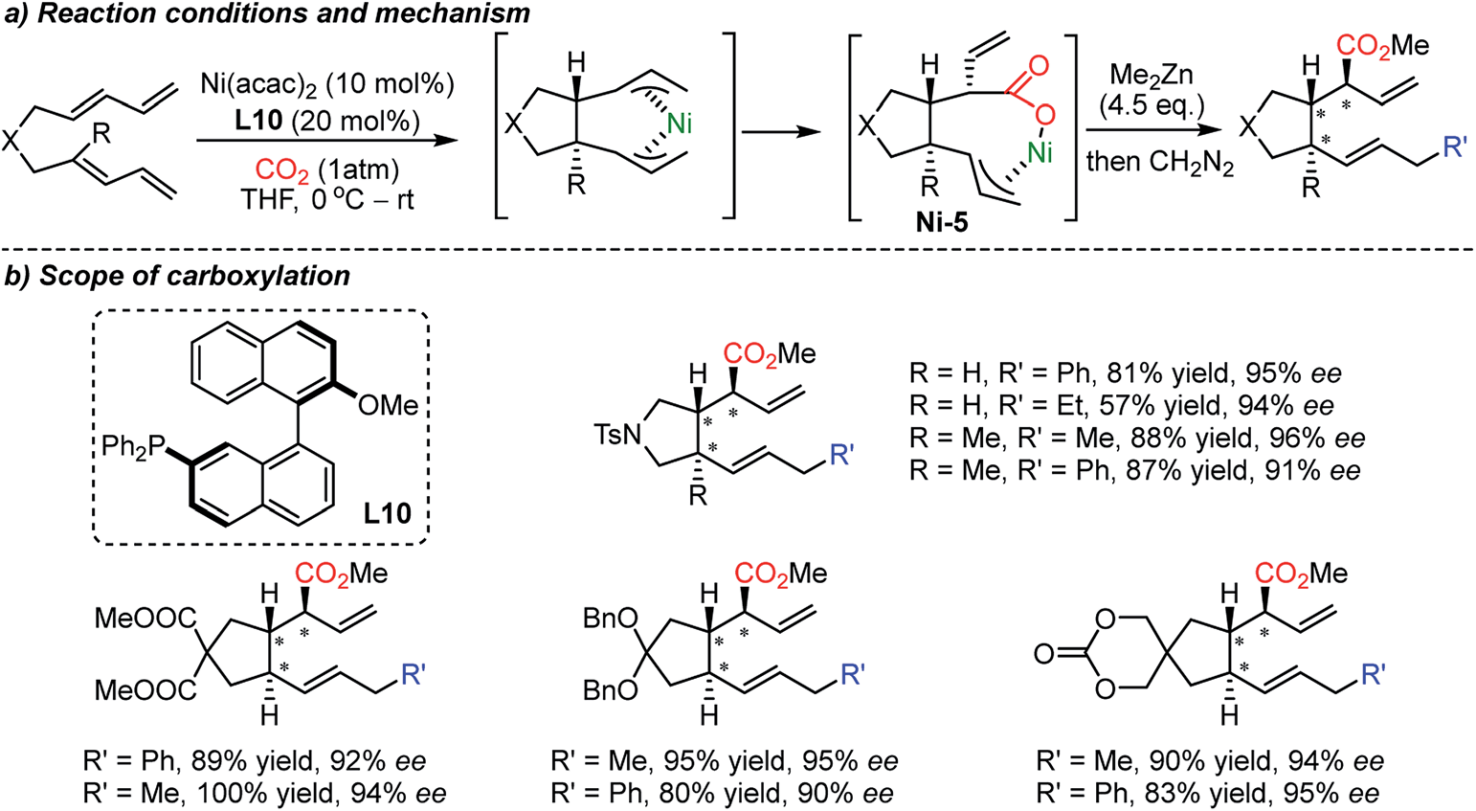
Asymmetric carboxylative cyclization of bis-1,3-dienes.

Besides the asymmetric synthesis of cyclic carbonates or polycarbonates with epoxides or diols,21b,^80^ the construction of enantioselective C–CO_2_ bonds using CO_2_ has been a formidable challenge under the influence of chiral catalysts or chiral environments. This is due to the high stability of CO_2_, limiting the scope of reaction partners; highly reactive organometallic species and/or harsh reaction conditions are necessary thus low stereoselectivity is in general expected.21*a* In 2004, the Mori group reported the carboxylative cyclization reaction of bis-1,3-dienes catalyzed by a Ni catalyst (Scheme 20).^81^ The authors performed facile 5-membered ring formation reactions in the presence of excess amounts of dialkyl zinc (4.5 equiv.). The obtained products possess three consecutive stereogenic centers with absolute diastereoselectivity with good yield and excellent enantioselectivity.

**Scheme 21:**
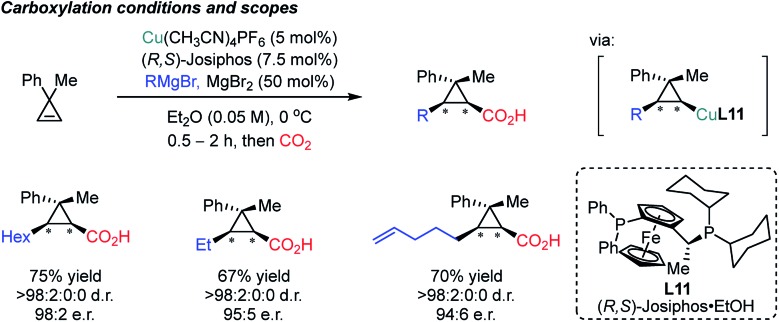
Asymmetric carbomagnesiation/carboxylation of cyclopropenes.

In 2017, the Marek group developed an enantioselective Cu-catalyzed carbomagnesiation reaction of cyclopropenes, which could be selectively carboxylated with CO_2_ as an electrophile (Scheme 21).^82^ High diastereoselectivity was observed which is not fully understood yet based on the control experiment without the copper catalyst (racemic but moderate diastereoselectivity, 9 : 1 dr). Other electrophiles such as iodine, bromine and allylbromide were smoothly incorporated to furnish the desired products. Although Grignard reagents are reactive nucleophiles, the sequential addition of the alkene and CO_2_ prevented direct attack of these nucleophiles on CO_2_ at low reaction temperature (0 °C) in the presence of a copper catalyst. The observed stereoselectivity was attributed to the stability of the stereogenic center at the carbon–Cu moiety, explaining the *cis* geometry between the nucleophile and electrophilic CO_2_.

**Scheme 22:**
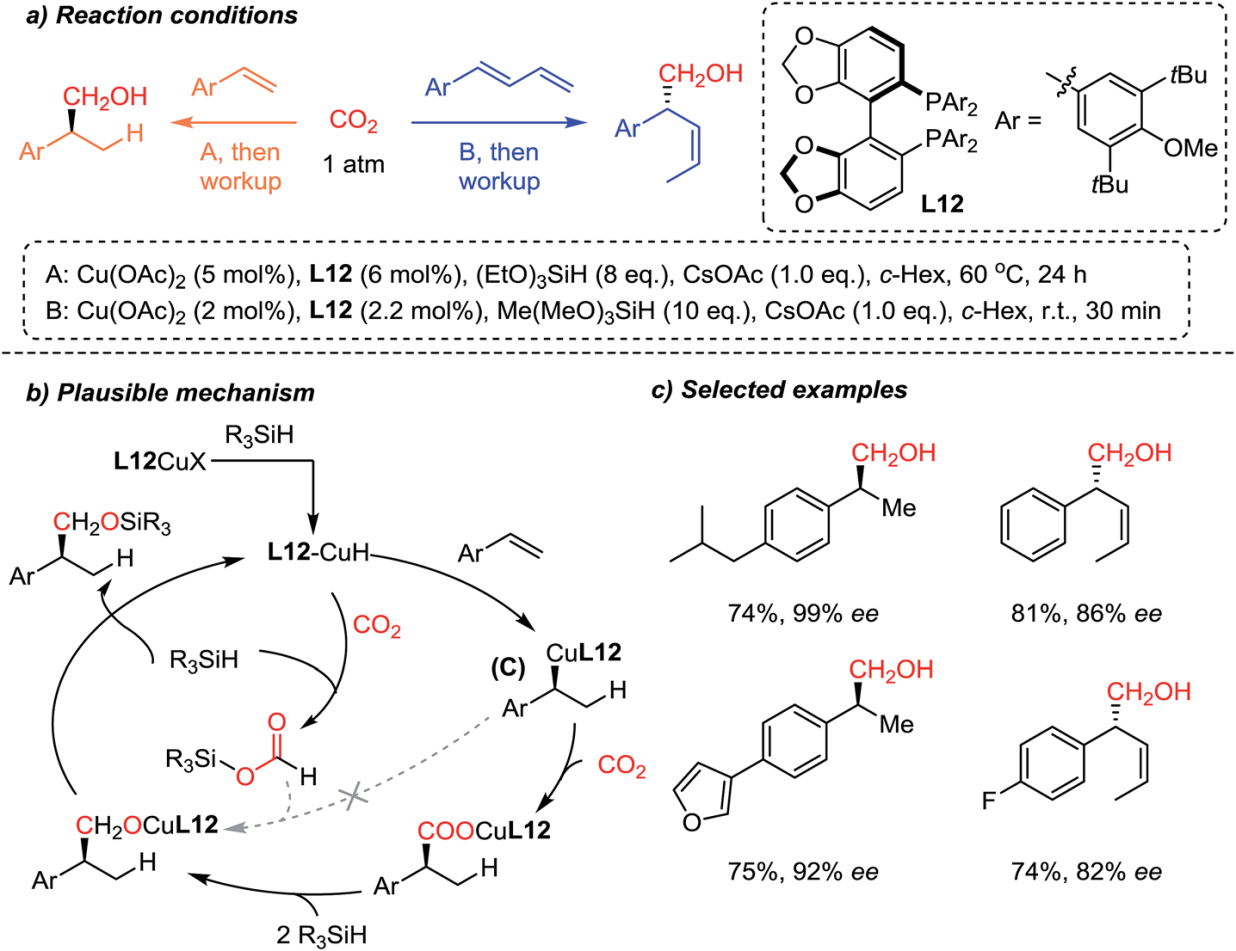
Enantioselective incorporation of CO_2_*via* hydrocarboxylation of styrene derivatives and dienes.

The Yu group^83^ recently reported a highly regio- and enantioselective copper-catalyzed CO_2_-functionalization reaction of olefins owing to enantioselective Cu–H catalysis^84^ (Scheme 22). Inspired by the CO_2_ reduction reaction to methanol^85^ and other higher alcohols,^86^ the authors developed the sequential enantioselective Cu–H addition, carboxylation and reduction reactions to achieve hydroxymethylation of olefins. A preliminary mechanistic study revealed that the **L12** Cu–R (**C**) species showed no reactivity toward reduced CO_2_ (R_3_Si–OCOH) (dashed arrow), indicating the direct carboxylation of **C** in the chiral environment to ensure the obtained high enantioselectivity. Furthermore, the developed methodology was applied to 1,3-dienes, affording (*Z*)-selective homoallylic alcohols with good enantioselectivities. Further derivatization of the hydroxymethylation products afforded elegant syntheses of enantioenriched (*R*)-(–)-curcumene^87^ and (*S*)-(+)-ibuprofen, starting from CO_2_ as a C1 building block.

Although asymmetric catalytic C–C bond formation has achieved relatively considerable progress,^88^ only a few methodologies have been reported with CO_2_ as a sustainable C1 source while creating stereogenic center(s) with high stereoselectivity. Considering that the carbon fixation process produces carbohydrates and biomass with absolute enantioselectivity, it is a logical extension to implement asymmetric carboxylation reactions in artificial CO_2_ fixation. Chemical synthesis offers various synthetic pathways and tools that can be easily tested, potentially providing a playground for facile screening and method development. For example, photochemical reactions with chiral catalysts including a chiral iridium catalyst^89^ or Lewis-acid assisted photocatalysis^90^ for CO_2_-functionalization are seemingly feasible methods to be developed. Considering the mode of action of RubisCo enzyme, redox-inactive metals and ligands (*e.g.* Mg–biotin complex) would be critical to improve the availability of CO_2_ in organic reactions.^91^ On the other hand, it could be inferred that chiral CO_2_-complexes may play a significant role in CO_2_-activation *via* bifunctional asymmetric catalysts.^92^ It would be exciting to see the development of CO_2_-functionalization, with foreseeable sustainability and increased utility of the final products in organic synthesis.

## 6. Conclusion and outlook: umpolung reactivities towards CO_2_

It is a formidable challenge to define an “ideal” carbon dioxide functionalization considering that many factors – environmental impact, atom-economy, sustainability, utility of products, and reaction conditions – are involved in designing reaction processes. Harnessing the full capacity of CO_2_-functionalization can be envisaged with sustainable and accessible chemical feed stocks, catalysts, and reaction conditions.

Victor Grignard, in 1912, stated this in his Nobel Lecture – “*Willstatter in fact recognized that …organic magnesium compounds must form and that the absorption of CO*_*2*_*gas by chlorophyll would in every way be comparable to the Grignard reaction*”. The mode of action of magnesium compounds in chlorophyll differs from what Grignard speculated, however, one of the earliest umpolung reactions with CO_2_ and Grignard reagents paved a way for modern CO_2_ functionalization to date. Considering the formation of Grignard reagents, an umpolung process utilized polarized bonds, C^*δ*+^–X^*δ*–^, by inverting the electronic nature of the carbon to nucleophilic by forming C^*δ*–^–Mg–X.

**Scheme 23:**
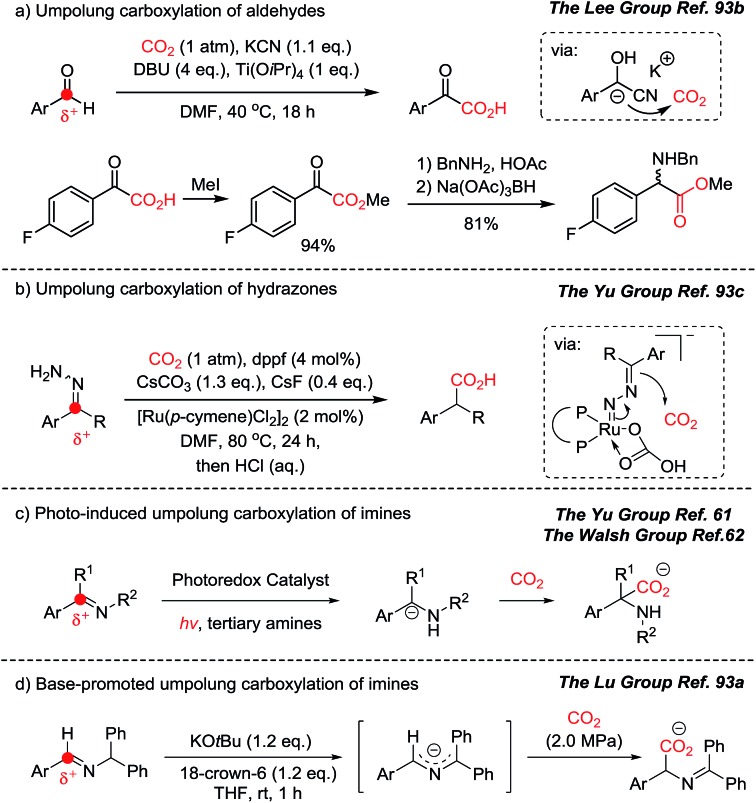
Direct umpolung carboxylation. (a) Umpolung carboxylation of aldehyde and reductive amination of α-keto acids to α-amino acids; (b) umpolung carboxylation of hydrazones; (c) photo-induced and (d) base-promoted umpolung carboxylation of imines.

In this context, recent developments in umpolung carboxylation reactions have shown unprecedented reaction patterns (Scheme 23):^61^,^62^,^93^ for example, umpolung reactivity has been implemented to functionalize CO_2_ for an aldehyde carboxylation reaction through a redox-neutral mechanism (Scheme 23a).93*b* The obtained product, α-keto acid, was smoothly converted to the corresponding α-amino acid under reductive amination reaction conditions mimicking the biosynthesis of various amino acids. The use of nitrogen-containing nucleophiles offers direct synthesis of α-amino acid derivatives (Scheme 23c–e). By employing cyanohydrin, hydrazone, photocatalysts, and a base, *in situ* generated umpolung species were transformed to the desired carboxylated products under mild reaction conditions, with or without reducing agents. This is particularly interesting to hypothesize the evolution of α-amino acids from the CO_2_-rich prebiotic environment. It has been postulated that cyanide is an abundant source of a carbon nucleophile in the synthesis of biologically active molecules in the primordial soup.^94^ The use of aldehydes, cyanide, and CO_2_ in synthesizing biologically active molecules is a promising step toward answering the important question: what is the origin of life? Was there an involvement of photochemical CO_2_-activation? Was it promoted by an optically pure component to induce homochirality? The forthcoming ideal CO_2_-functionalization may answer these conundrums.

In summary, this *Perspective* collects the recent literature in CO_2_ functionalization and groups it into four categories: (1) metal catalyzed direct carboxylation, (2) photocatalytic carboxylation reactions, (3) redox-neutral carboxylation, and (4) asymmetric introduction of catalytic CO_2_ for C–C bond formation reactions. Even a broad scope of substrates and remote site functionalization were achieved; transition metal-catalyzed reductive carboxylation is mostly limited to CO_2_ insertion reaction with (over)stoichiometric amounts of reducing reagents. However, photoredox catalysts present promising access to more diversified CO_2_ reactions, like dicarbofunctionalization, single-electron reduction and radical coupling *via* a redox-neutral mechanism. Thanks to these developments of methodologies, as discussed at the end of Section 5, more examples in challenging enantioselective C–CO_2_ bond formation will be realized in the near future. Although enzymatic CO_2_ functionalization reactions are not covered in this *Perspective*, they have shown their very promising application in artificial carbon recycling processes.^51^,^95^ Synergetic and interdisciplinary CO_2_ fixation with biological and chemical catalysts will be particularly interesting in (asymmetric) photocatalytic conversion of CO_2_, truly mimicking photosynthesis to provide ideal CO_2_ functionalization reactions.

## Conflicts of interest

There are no conflicts to declare.
